# Health‐seeking behaviors of patients with acute respiratory infections during the outbreak of novel coronavirus disease 2019 in Wuhan, China

**DOI:** 10.1111/irv.12804

**Published:** 2020-09-10

**Authors:** Juan Yang, Hui Gong, Xinhua Chen, Zhiyuan Chen, Xiaowei Deng, Mengcen Qian, Zhiyuan Hou, Marco Ajelli, Cecile Viboud, Hongjie Yu

**Affiliations:** ^1^ School of Public Health Fudan University, Key Laboratory of Public Health Safety, Ministry of Education Shanghai China; ^2^ Department of Epidemiology and Biostatistics Indiana University School of Public Health Bloomington IN USA; ^3^ Division of International Epidemiology and Population Studies Fogarty International Center National Institutes of Health Bethesda MD USA

**Keywords:** acute respiratory infections, China, health‐seeking behaviors, novel coronavirus disease 2019

## Abstract

We conducted two surveys to evaluate the health‐seeking behaviors of individuals with acute respiratory infections (ARI) during the COVID‐19 outbreak in Wuhan, China. Among 351 participants reporting ARI (10.3%, 351/3,411), 36.5% sought medical assistance. Children were more likely to seek medical assistance than other age‐groups (66.1% vs. 28.0%‐35.1%). This population‐based study demonstrates that the majority of patients with ARI symptoms did not seek medical assistance during the COVID‐19 outbreak in Wuhan. These findings may be used to refine the estimates of disease burden and clinical severity of COVID‐19 and to plan for health resources allocation.

## 1 INTRODUCTION

The novel coronavirus disease 2019 (COVID‐19) emerged in Wuhan, Hubei province, China in December 2019 and rapidly spread across the world.^1^, ^2^ During emergency situations, rapid measurements of changes in health‐seeking behavior are critical to understand healthcare utilization. Moreover, estimating the disease burden and clinical severity of COVID‐19 is key to identify appropriate intervention strategies and allocate healthcare resources. However, quantifying these metrics while a pandemic is unfolding is challenging due to limitations in passive surveillance. Here, we assess the health‐seeking behavior of residents with acute respiratory infections (ARI) during the outbreak of COVID‐19 between December 2019 and March 2020 in Wuhan, China.

## 2 METHODS

Between March 10 and 24, 2020, two population‐based surveys were conducted in Wuhan to understand the health‐seeking behaviors of patients suffering from ARI (presence of fever and/or any respiratory symptoms, eg, cough and sore throat) during the COVID‐19 outbreak. The surveys included the following: (a) a telephone‐and‐online survey of 2,595 adults (individuals aged ≥18 years); (b) an online survey of 816 children (individuals aged <18 years). The study participants were current residents who had lived in Wuhan for at least three months before the date of the survey. Based on the assumption that 6% of the population would have ARI,^3^ we calculated that a minimum sample size of 784 participants per age‐group (ie, 3‐17, 18‐39, 40‐59, and 60 years of age) would allow a health care‐seeking proportion of 60% to be estimated, with a statistical significance level of 5%, and 14% of marginal error.

**Figure 1:**
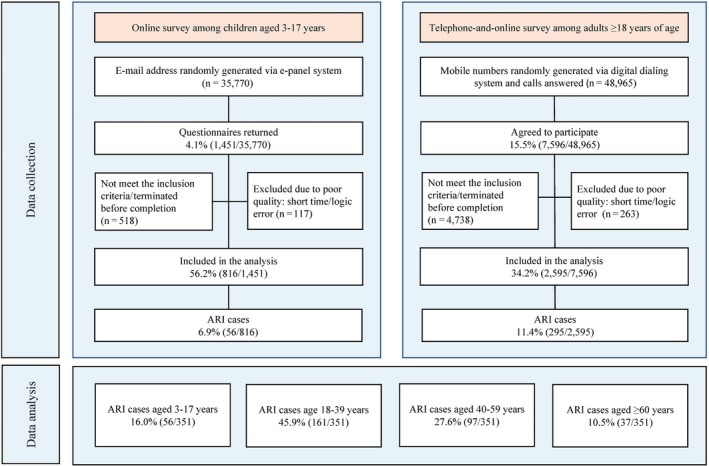
Flowchart of telephone and online surveys. The percentage shows the proportion of persons between two adjacent stages. The denominators are the number of persons in the former stage

The telephone‐and‐online survey among adults was conducted in two steps. First, we randomly dialed Wuhan mobile phone numbers to invite the participants through a computer‐assisted interviewing system. An overview of the study was provided at the beginning of each call. We attempted to contact each generated number twice a day at different hours and one more time on the following day; if no contact was established after the third call, the number was classified as invalid or unreachable. Second, we sent a telephone message with the online link of our questionnaire to the identified participants, who were asked to complete the questionnaire on their own. Of 48 965 calls answered, 2595 persons (5.3%) completed the questionnaires and included in the analysis (Figure 1).

The online survey of children was commissioned to the ePanel data company, which has a national online survey library with emails and household information registered. An online link to our questionnaire was provided to Wuhan families with children. The questionnaire was answered by the parents of recruited children. Among 35 770 email invitations, 816 persons (2.3%) completed the questionnaires and included in the analysis (Figure 1).

Random dials/emails with proportional quota sampling (female‐to‐male 1:1) were used to ensure that respondents were demographically representative of the population who owns a mobile phone. Characteristics of respondents were collected, including age and sex. We also obtained the history of ARI for respondents in the last three months, and whether they sought medical assistance for these symptoms. Questionnaires completed in less than 2 minutes and containing logical errors were excluded from the analysis (Figure 1). The questionnaires are reported in Appendix. The surveys were approved by the Institutional review board from School of Public Health, Fudan University (IRB#2020‐01‐0801). Informed consent was obtained from all respondents/parents of children before surveys.

To estimate the proportion of individuals with ARI and their probability of seeking medical assistance, participants’ ages were weighted to match the age structure of the Wuhan population who owns a mobile phone and has access to the Internet.^4^ Chi‐square test and Fisher's exact test were used for binary variables. Two‐sided *P* values <.05 were considered to indicate statistical significance. Binomial distributions were used to estimate the 95% CIs for binary variables.

## 3 RESULTS

**Figure 2:**
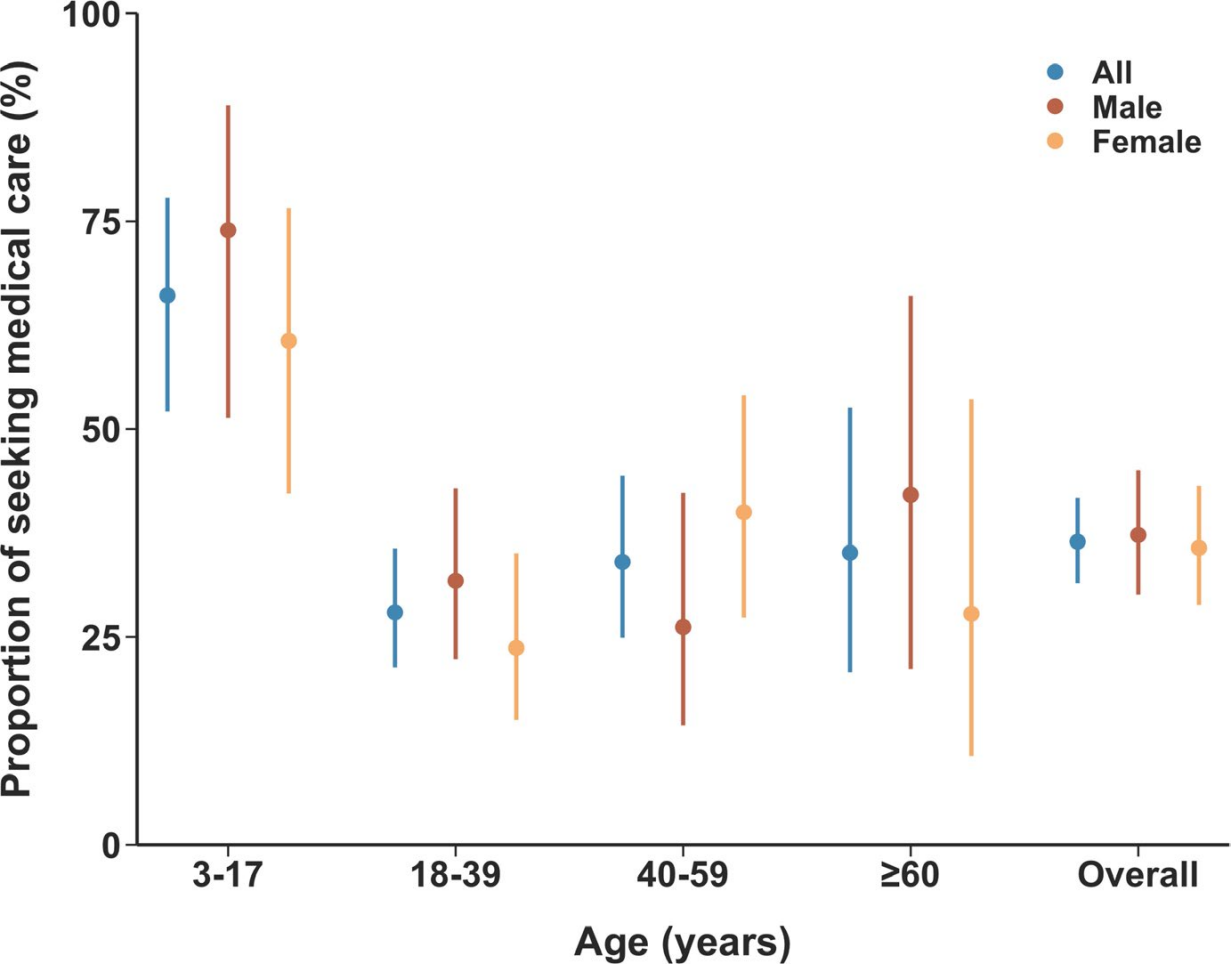
Proportion of study participants with acute respiratory infections seeking medical assistance

A total of 3,411 study participants were included in our analysis. 49.6% (1691/3411) were female. Of them, 351 participants (10.3%, 95% CI 9.3%‐11.4%) reported ARI during the COVID‐19 outbreak in Wuhan, with higher proportions of ARI observed in adults than children (9.3%‐12.5% depending on the age‐group vs. 6.9%, χ^2^ = 17.57, *P* < .001). Among ARI cases, 36.5% (95% CI 31.5%‐41.8%) sought medical care. No significant difference of health‐seeking behaviors was observed between male and female (Figure 2). Children were more likely to seek medical assistance than other age‐groups (66.1% vs. 28.0%‐35.1%, χ^2^ = 26.50, *P* < .001) (Table). Adjusting for the age structure of the Wuhan population who owns a mobile phone and has access to the Internet,^4^ the overall proportion of patients with ARI was 10.5% (95% CI 9.4%‐11.6%), and the adjusted proportion of seeking medical assistance for ARI was 39.0% (95% CI 32.3%‐46.7%).

A small proportion of patients (16.4%, 95% CI 10.7%‐24.2%) with ARI sought medical care in private clinics, while around 30% visited community‐based health service centers or different types of hospitals. Among children who did not seek medical care, 54.5% (95% CI 22.9%‐82.9%) reported to be afraid of acquiring COVID‐19 when visiting a hospital. Among adults who did not seek medical care, 51.6%‐54.2% reported mild illness as a reason for their behavior (Table).

## 4 DISCUSSION

The study provides valuable insights in the health‐seeking behaviors of ARI patients during the COVID‐19 outbreak in Wuhan. Although adults were more likely to report ARI than children aged 3‐17 years, a smaller proportion of adults sought medical assistance. Among the reasons for not seeking medical care, parents reported concerns with COVID‐19 infections for their sick children when visiting a hospital, while experiencing mild illness was linked to lack of seeking care in adults.

This study analyzed the health‐seeking behaviors of ARI patients during COVID‐19 outbreak in Wuhan, with 35.4% of ARI patients seeking medical assistance, which was lower than that during 2009 influenza pandemic (46.1% for ARI cases),^3^ and much lower than that in the absence of any pandemics (54.1%‐79.7% for influenza‐like‐illness cases in 2017/2018 influenza season).^5^ Over half of ARI cases in our study did not seek medical help due to mild illness.

Between January 24 and February 10, 2020, an active community universal screenings of fever cases was conducted among general population in Wuhan.^6^ Patients with confirmed or highly suspected COVID‐19 were sent to the designated sites for treatment. Suspected cases with fever were kept in the fever clinics for medical observation. Mild cases with fever who have not been identified as suspected patients were moved to designated sites for centralized isolation and medical observation. Cases with fever but negative for SARS‐CoV‐2 stayed at home for observation.^6^ The control strategy for COVID‐19 accordingly contributed to the lower probability of seeking medical help.

An increasing amount of attentions have been paid to the routine medical service for other patients (eg, those affected by non‐communicable diseases) during the COVID‐19 pandemic, which was disrupted due to lockdown policy, reduction in transport links, postponement of routine medical appointments and tests, and predicted increased risk of COVID‐19 infection during medical visits, etc.The WHO conducted a rapid assessment survey of service delivery for non‐communicable diseases during the COVID‐19 pandemic. Around half of the countries surveyed have partially or completely disrupted services for hypertension, cancer, diabetes, and diabetes‐related complications.^7^ In Wuhan, nearly half of the patients with breast cancer during the COVID‐19 outbreak had to discontinue or modify their schedules of necessary anticancer treatments.^8^ Our analysis revealed that the health‐seeking behaviors of ARI patients were also affected during the COVID‐19 outbreak in Wuhan, lower than that during 2009 influenza pandemic,^3^ and much lower than that in the absence of any pandemics.^5^ Using a non‐specific case definition of ARI (including infectious and non‐infectious conditions), this study enabled us to reveal the extensive impact of pandemic on health‐seeking behaviors of individuals with specific symptoms instead of with a specific disease. These findings can be instrumental to plan for health resources allocation.

A range of studies has quantified the clinical severity of COVID‐19, particularly as regards the case and infection fatality ratios.^9^, ^10^, ^11^, ^12^ However, these estimates were mainly based on laboratory‐confirmed cases captured by surveillance system, which may overestimate COVID‐19 severity. In fact, we found that the majority of mild cases did not seek medical assistance and thus were hardly detectable by surveillance systems. The number of COVID‐19 cases in the community remains unclear due to changes in health‐seeking behaviors during the outbreak; an assessment of the full severity pyramid of COVID‐19 is still lacking. While several studies have assessed the healthcare demand of the COVID‐19 pandemic in terms of hospital beds, ICU beds, and ventilators,^13^, ^14^ no study has evaluated the demand on outpatient facilities. Our study could provide key metrics that can be instrumental to fill this gap.

However, it is important to stress that the health‐seeking behavior of patients with ARI may differ from that of COVID‐19 cases. We asked adult participants if they were diagnosed as laboratory‐confirmed or probable COVID‐19 cases. Only 2.2% of them (57/2,595) self‐reported to be a laboratory‐confirmed or probable COVID‐19 case. Among COVID‐19 patients, 35.1% (20/57) did not answer the questions regarding health care‐seeking behavior. Hence, the small sample size did not allow us to compare health care‐seeking behavior between COVID‐19 cases and those with ARI. As compared to a study conducted in Wuhan, during the 2009 H1N1 influenza pandemic,^3^ we found a larger proportion of ARI cases in the study populations (10.3% vs. 4.9%). This suggests that a faction of our ARI cases may be attributed to SARS‐CoV‐2 infections (also in light of the low circulation of other ARI‐associated pathogens such as influenza virus during the study period^15^).

Our study had several limitations. First, for the telephone‐and‐online survey among adults, the study participants were those who own a mobile phone and have access to the Internet. While for the online survey of children, the study participants were from Wuhan families with emails. The families with better education background and higher income are more likely to have emails. Accordingly, the health‐seeking behaviors could not represent that of the whole population in Wuhan. Using the age profile of the population who owns a mobile phone and has access to the Internet, we could partially adjust the probability of health‐seeking behaviors. Second, the low response rate in our survey may lead to a potential bias toward individuals who paid more attention to health and thus were more likely to participate in our study. This may have led to overestimate the probability of health‐seeking medical assistance. We were unable to evaluate the representativeness of the study participants since the demographic characteristics of those who did not participate in the present study were not available. Although we adjusted the probability of seeking medical care using the actual age structure of Wuhan population who owns a mobile phone and has access to the Internet to minimize the bias, factors like socio‐economic status, medical insurance, and education level were not accounted for. Accordingly, it should be prudent to generalize study findings to the whole population of Wuhan. Third, as it was a retrospective study, and the ARI illness and the corresponding health‐seeking behaviors were all self‐reported, recall bias was inevitable and was difficult to be quantified.

In conclusion, this study demonstrates that the majority of patients with ARI symptoms did not seek medical assistance during the COVID‐19 outbreak in Wuhan. These findings may be used to refine the estimates of disease burden and clinical severity of COVID‐19, and the health demand associated with this pandemic, and to plan for health resources allocation.

## CONFLICT OF INTEREST

MA has received research funding from Seqirus and HY from Sanofi Pasteur, GlaxoSmithKline, Yichang HEC Changjiang Pharmaceutical Company, and Shanghai Roche Pharmaceutical Company. None of those research funding is related to COVID‐19. All other authors report no competing interests.

## AUTHOR CONTRIBUTION

**Juan Yang:** Data curation (lead); Formal analysis (lead); Methodology (lead); Project administration (lead); Supervision (lead); Writing‐original draft (lead). **Gong Hui:** Formal analysis (lead); Investigation (supporting). **Chen Xinhua:** Formal analysis (lead); Investigation (supporting). **Chen Zhiyuan:** Formal analysis (supporting); Visualization (supporting). **Deng Xiaowei:** Formal analysis (supporting). **Qian Mengcen:** Investigation (lead). **Hou Zhiyuan:** Investigation (lead). **Ajelli Marco:** Methodology (supporting); Writing‐review & editing (supporting). **Cecile Viboud:** Methodology (supporting); Writing‐review & editing (supporting). **Hongjie Yu:** Conceptualization (lead); Funding acquisition (lead); Methodology (lead); Supervision (lead); Writing‐review & editing (lead).

5

**Table 1:** Health‐seeking behaviors of residents with acute respiratory infections during the COVID‐19 pandemic in Wuhan, stratified by age‐groups

	All		3‐17 years		18‐39 years		40‐59 years		≥60 years		Chi‐square test	P value
	No.	Proportion (%)^a^	No.	Proportion (%)^a^	No.	Proportion (%)^a^	No.	Proportion (%)^a^	No.	Proportion (%)^a^		
Acute respiratory infections	351	10.3 (9.3, 11.4)	56	6.9 (5.3, 8.9)	161	12.5 (10.7, 14.4)	97	10.7 (8.8, 12.9)	37	9.3 (6.7, 12.8)	17.57	<0.001
Proportion of seeking medical assistance of ARI cases	128	36.5 (31.5, 41.8)	37	66.1 (52.1, 77.8)	45	28.0 (21.3, 35.7)	33	34.0 (24.9, 44.4)	13	35.1 (20.7, 52.6)	26.50	<0.001
Medical attendance at different health institutions^b^											6.96^f^	0.541
Private clinics	21	16.4 (10.7, 24.2)	9	24.3 (12.4, 41.6)	7	15.6 (7.0, 30.1)	3	9.1 (2.4, 25.5)	2	15.4 (2.7, 46.3)		
Community health service centers	34	26.6 (19.3, 35.2)	10	27.0 (14.4, 44.4)	10	22.2 (11.7, 37.5)	9	27.3 (13.9, 45.8)	5	38.5 (15.1, 67.7)		
County/district hospitals	37	28.9 (21.4, 37.7)	8	21.6 (10.4, 38.7)	17	37.8 (24.2, 53.5)	7	21.2 (9.6, 39.4)	5	38.5 (15.1, 67.7)		
Municipal hospitals	43	33.6 (25.6, 42.5)	11	29.7 (16.4, 47.2)	14	31.1 (18.6, 46.8)	14	42.4 (26.0, 60.6)	4	30.8 (10.4, 61.1)		
Provincial hospitals	35	27.3 (20.0, 36.1)	11	29.7 (16.4, 47.2)	15	33.3 (20.4, 49.1)	7	21.2 (9.6, 39.4)	2	15.4 (2.7, 46.3)		
Outpatient visit^c^	88	68.8 (59.9, 76.5)	21	56.8 (39.6, 72.5)	34	75.6 (60.1, 86.6)	27	81.8 (63.9, 92.4)	6	46.2 (20.4, 73.9)	Fisher's exact test	0.029
Hospitalization^c^	40	31.3 (23.5, 40.1)	16	43.2 (27.5, 60.4)	11	24.4 (13.4, 39.9)	6	18.2 (7.6, 36.1)	7	53.8 (26.1, 79.6)		
ICU admission^d^	18	45.0 (29.6, 61.3)	10	62.5 (35.9, 83.7)	4	36.4 (12.4, 68.4)	0	0 (0, 48.3)	4	57.1 (20.2, 88.2)	Fisher's exact test	0.054
Reasons for no medical attendance^e^											Fisher's exact test^g^	0.050
Mild illness	111	51.6 (43.2, 60.0)	3	27.3 (7.5, 63.5)	62	53.4 (42.0, 64.5)	33	51.6 (36.5, 66.3)	13	54.2 (30.7, 76.0)		
Fear of acquiring COVID‐19 when visiting a hospital	63	29.3 (22.2, 37.6)	6	54.5 (22.9, 82.9)	30	25.9 (17.1, 37.1)	21	32.8 (20.3, 48.4)	6	25 (9.8, 50.5)		
Hospital suspension during the epidemic	15	7.0 (3.7, 12.6)	2	18.2 (3.8, 55.6)	6	5.2 (2.0, 13.0)	2	3.1 (0.6, 13.9)	5	20.8 (7.4, 46.3)		
Other	26	12.1 (7.6, 18.7)	0	0 (0, 36.2)	18	15.5 (8.9, 25.6)	8	12.5 (5.4, 26.2)	0	0 (0, 20.6)		

^a^ Mean, 95% CI.

^b^ This is a multiple‐choice question answered by acute respiratory infection cases with medical attendance.

^c^ Among medically attended cases.

^d^ Among hospitalized cases.

^e^ In children aged 3‐17 years, 8 out of 19 acute respiratory infection cases who did not seek medical assistance did not answer this question.

^f^ 40‐59 and ≥ 60 years were aggregated for chi‐square test due to small sample size.

^g^ 18‐39, 40‐59, and ≥ 60 years were aggregated for chi‐square test due to small sample size.

## Supporting information

Supplementary Material
