# Supplementary material for: Health‐seeking behaviors of patients with acute respiratory infections during the outbreak of novel coronavirus disease 2019 in Wuhan, China
Source: Influenza Other Respir Viruses. 2020 Sep 10;15(2):188–94. doi: 10.1111/irv.12804 (PMC7902258; doi:10.1111/irv.12804)
Supplement: Supplementary file 1 — Supplementary Material [file IRV-15-188-s001.docx]

**Supplementary Appendix**

**Health seeking behaviors of residents with acute respiratory infections during the outbreak of novel coronavirus disease 2019 in Wuhan, China**

Juan Yang, PhD, Hui Gong, BSc, Xinhua Chen, BSc, Zhiyuan Chen, BSc, Xiaowei Deng, MSc, Mengcen Qian, PhD, Zhiyuan Hou, PhD, Marco Ajelli, PhD, Cecile Viboud, PhD, Prof Hongjie Yu, PhD

## Questionnaires

### Online survey among children

**Part 1. Case identification**

1.Where are you currently living?

| a. Wuhan | b. Shanghai | c. None of the above |
| --- | --- | --- |

If the answer is “none of the above”, the interview ends.

2.Do you have children?

| a. Yes | b. No |
| --- | --- |

If the answer is "no", the interview ends.

3. How old is your child? (if there are more than one child aged 3-17, please choose one child and answer all the following questions.)

| a. 3-5 yrs | b. 6-9 yrs | c. 10-14 yrs |
| --- | --- | --- |
| d. 15-17 yrs | e. None of the above |  |

If the answer is “none of the above”, the interview ends.

4. What’s the sex of your child?

| a. Female | b. Male |
| --- | --- |

5. What is the relationship with your child?

| a. Mother | b. Father | c. Other |
| --- | --- | --- |

If the answer is “other”, the interview ends.

**Part 2. Treatment status of children**

1. Have your child had any fever and/or respiratory symptoms (e.g., cough, sore throat) during the epidemic of COVID-19 in Wuhan from last December through to March 2020?

| a. Yes | b. No |
| --- | --- |

2. During the epidemic of COVID-19 in Wuhan from last December through to March 2020, did your child seek medical treatment for the symptoms mentioned above? (multiple choice)

| a. Was isolated at home without medication, under observation | b. Self-medicated |
| --- | --- |
| c. Sought medical advice online | d. Visited private clinics |
| e. Visited community-based health service centers | f. Visited county/district hospitals |
| g. Visited municipal hospitals | h. Visited provincial hospitals |
| i. Other |  |

If the subject only chooses one of a, b, or c, then skip to question 5. If the subject' s answer includes any of d, e, f, g, or h, question 5 is not required.

3. What kind of treatment has your child obtained for the above diseases?

| a. Outpatient visit only | b. Hospitalization only | c. Both outpatient visit and hospitalization |
| --- | --- | --- |

4. During the epidemic of COVID-19 in Wuhan from last December through to March 2020, has your child been admitted to the ICU due to the above symptoms?

| a. Yes | b. No |
| --- | --- |

5.What is the main reason that your child didn’t go to medical institutions after onset of the above symptoms?

| a. Mild illness |
| --- |
| b. Fear of acquiring COVID-19 when visiting a hospital |
| c. Hospital suspension during the epidemic |
| d. Other |

### Telephone-and-online survey among adults

1.Are you currently living in Wuhan (Or have you been living in Wuhan for the past 3 months)?

| a. Yes | b. No |
| --- | --- |

If the answer is "no", the interview ends.

2. What’s your age?

| a. 18-24 yrs | b. 25-39 yrs | c. 40-59 yrs |
| --- | --- | --- |
| d. ≥60 yrs | e. None of the above |  |

If the answer is “none of the above”, the interview ends.

3. What’s your sex?

| a. Female | b. Male |
| --- | --- |

4. Have you had any fever and/or respiratory symptoms (e.g., cough, sore throat) during the epidemic of COVID-19 in Wuhan from last December through to March 2020?

| a. Yes | b. No |
| --- | --- |

5. During the epidemic of COVID-19 in Wuhan from last December through to March 2020, did you seek medical treatment for the symptoms mentioned above? (multiple choice)

| a. Was isolated at home without medication, under observation | b. Self-medicated |
| --- | --- |
| c. Sought medical advice online | d. Visited private clinics |
| e. Visited community-based health service centers | f. Visited county/district hospitals |
| g. Visited municipal hospitals | h. Visited provincial hospitals |
| i. Other |  |

If the subject only chooses one of a, b, or c, then skip to question 8. If the subject' s answer includes any of d, e, f, g, or h, question 8 is not required.

6. What kind of treatment have you obtained for the above diseases?

| a. Outpatient visit only | b. Hospitalization only | c. Both outpatient visit and hospitalization |
| --- | --- | --- |

7. During the epidemic of COVID-19 in Wuhan from last December through to March 2020, have you been admitted to the ICU due to the above symptoms?

| a. Yes | b. No |
| --- | --- |

8. What is the main reason that you didn’t seek medical care after onset of the above symptoms?

| a. Mild illness |
| --- |
| b. Fear of acquiring COVID-19 when visiting a hospital |
| c. Hospital suspension during the epidemic |
| d. Other |
